# A case report of coronary artery aneurysms with restenosis and stent fractures developed 14 years after sirolimus eluting stents implantation successfully treated with drug-coated balloons

**DOI:** 10.1093/ehjcr/ytae050

**Published:** 2024-01-29

**Authors:** Takanari Fujita, Junichi Tazaki, Mamoru Toyofuku

**Affiliations:** Department of Cardiovascular Medicine, Japanese Red Cross Wakayama Medical Center, 4-20 Komatsubara-dori, Wakayama 640-8558, Japan; Department of Cardiovascular Medicine, Japanese Red Cross Wakayama Medical Center, 4-20 Komatsubara-dori, Wakayama 640-8558, Japan; Department of Cardiovascular Medicine, Japanese Red Cross Wakayama Medical Center, 4-20 Komatsubara-dori, Wakayama 640-8558, Japan

**Keywords:** Case report, Coronary artery aneurysm, Percutaneous coronary intervention, Drug-coated balloon, Drug-eluting stent, In-stent restenosis

## Abstract

**Background:**

Coronary aneurysms following drug-eluting stent implantation are rare but associated with adverse events.

**Case summary:**

An 80-year-old male admitted to our hospital with resting chest discomfort. He had undergone percutaneous coronary interventions (PCIs) with first-generation sirolimus-eluting stent (SES) implantation to the right coronary artery (RCA) and left anterior descending artery (LAD) 14 years ago. Coronary angiography revealed coronary aneurysms and stent fractures in the RCA and LAD where SES was implanted. The aneurysm sizes of the RCA and LAD were 7 × 8 and 7 × 10 mm, respectively. Moreover, in-stent restenosis (ISR) with ischaemia were found in the LAD. The patient was at high risk for cardiac surgery and the coronary aneurysms were not suitable for percutaneous interventions. Therefore, we treated only ISR lesions using drug-coated balloons (DCBs) without intervention for coronary aneurysms. Intravascular ultrasound (IVUS) revealed that the first guide wire went outside the malapposed stents. After rewiring using a double-lumen microcatheter with another guide wire, IVUS confirmed the second guide wire passed entirely inside the stents. Then, the ISR lesions were dilated with high-pressure balloons and DCBs. The post-procedural course was uneventful and his symptoms were relieved.

**Discussion:**

This case demonstrated coronary aneurysms with ISR and stent fractures 14 years after SES implantation. Depending on patient background and lesion morphology, DCB can be one of the treatment options. Intravascular imaging is useful to guide PCI in patients with coronary aneurysms.

**Learning points**

- Coronary aneurysm following drug-eluting stent implantation is rare but diagnosed in patients with in-stent restenosis.
- Drug-coated balloon can be one of the treatment options for in-stent restenosis associated with coronary aneurysms.
- Intravascular imaging is useful to guide percutaneous coronary intervention in patients with coronary aneurysms.

## Introduction

The incidence of coronary aneurysms following drug-eluting stent (DES) implantation has been reported 0.2–2.3%.^1^ The proposed underlying mechanism includes vessel injury during the initial procedure, hypersensitivity reaction to DES components, and infection.^2^ Although coronary aneurysms are usually asymptomatic and may even resolve spontaneously, it is associated with adverse clinical events. While thrombosis and rupture are life-threatening problems, coronary aneurysms may also be detected in patients who have developed restenosis.

## Summary figure

**Figure:**
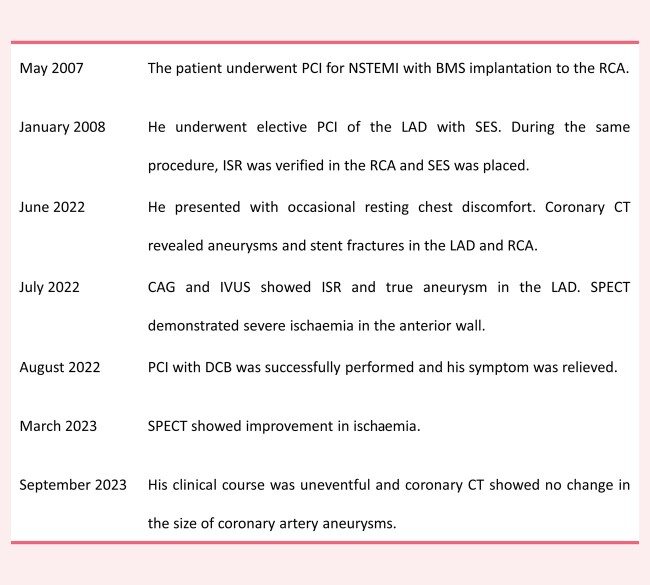


## Case presentation

**Figure 1:**
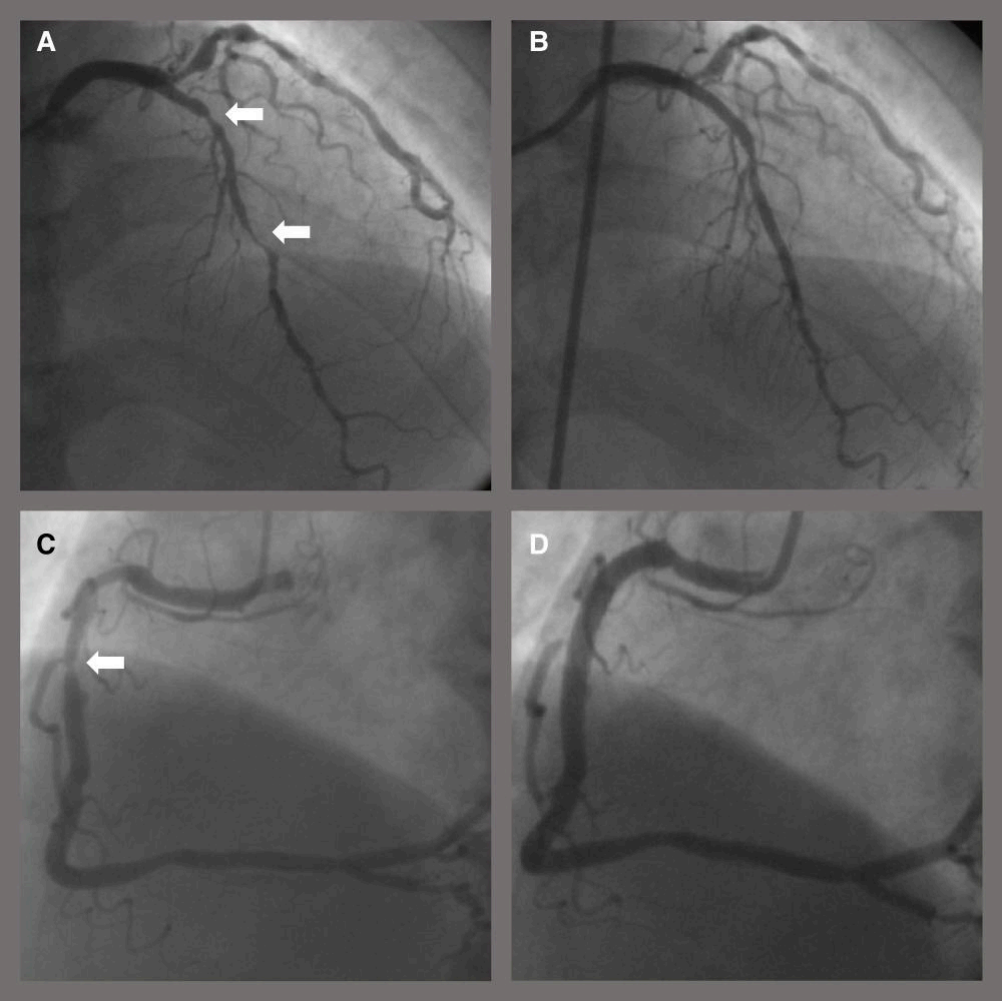
Coronary angiography and stenting 14 years before. (*A*) Left anterior descending artery *de novo* lesions (arrows). (*B*) Two sirolimus-eluting stents were successfully implanted. (*C*) Bare metal stent restenosis (arrow) in the right coronary artery. (*D*) Two sirolimus-eluting stents were successfully implanted.

An 80-year-old male admitted to our hospital due to occasional resting chest discomfort. He had undergone percutaneous coronary intervention (PCI) for non-ST-segment elevation myocardial infarction with bare metal stent implantation to the proximal right coronary artery (RCA). Eight months after the first procedure, he underwent elective PCI of the proximal and medial left anterior descending artery (LAD) with sirolimus-eluting stent (SES) (*Figure 1A* and *B*). During the same procedure, in-stent restenosis (ISR) was verified in the RCA and SES was placed (*Figure 1C* and *D*). He remained asymptomatic for 14 years until this time.

He also had a medical history of distal cholangiocarcinoma and had undergone pancreatoduodenectomy 2 months before. His physical function declined significantly after the surgery.

**Figure 2:**
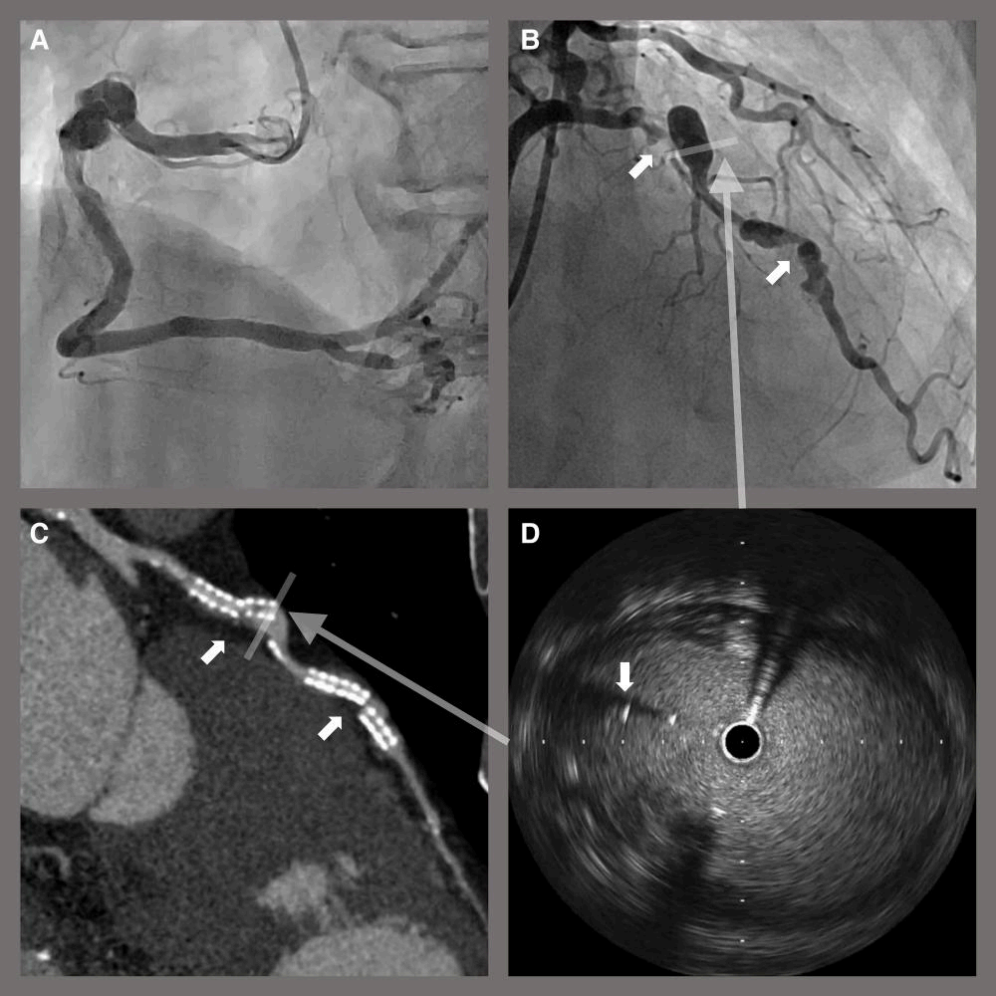
(*A*) Right coronary angiography revealed multiple aneurysm formation in sirolimus-eluting stent implanted site. (*B*) Left coronary angiography also showed multiple aneurysm formation in sirolimus-eluting stent implanted site. Moreover, restenosis and stent fractures (arrows) were observed. The grey line perpendicular to the vessel at the end of the long grey arrow indicates the area where the intravascular ultrasound image in *D* was obtained. (*C*) Computed coronary angiography of left anterior descending artery showed stent fractures (arrows). The grey line perpendicular to the vessel at the end of the long grey arrow indicates the area where the intravascular ultrasound image in *D* was obtained. (*D*) Intravascular ultrasound images showed true aneurysm with a maximum size of 7 × 10 mm at the distal end of the proximal sirolimus-eluting stent in the left anterior descending artery. An arrow indicates the stent.

On examination, his blood pressure was 122/79 mmHg, with a regular pulse rate of 85 b.p.m. A grade 2 diastolic murmur was present on auscultation. High-sensitivity troponin I was within normal range, and N-terminal pro-brain natriuretic peptide was 412 pg/mL. The electrocardiogram showed normal sinus rhythm without ST-T segment abnormality. Transthoracic echocardiography showed left ventricular inferior wall hypokinesis with preserved ejection fraction and moderate aortic regurgitation. Coronary computed tomography angiography revealed coronary aneurysms and stent fractures in the RCA and LAD (*Figure 2C*). The aneurysm sizes of the RCA and LAD were 7 × 8 and 7 × 10 mm, respectively. Stress perfusion single-photon emission computed tomography (SPECT) demonstrated severe ischaemia at the anterior wall. Coronary angiography showed coronary aneurysms and stent fractures in the RCA and LAD where SES was implanted (*Figure 2A* and *B*). Moreover, ISRs were observed in the LAD. Intravascular ultrasound (IVUS) revealed that the aneurysms were true aneurysm morphology (*Figure 2D*). The patient was at high risk for cardiac surgery and the coronary aneurysms were not suitable for percutaneous interventions. After heart team discussion and consultation with the patient and his family, we planned treatment with drug-coated balloons (DCBs).

**Figure 3:**
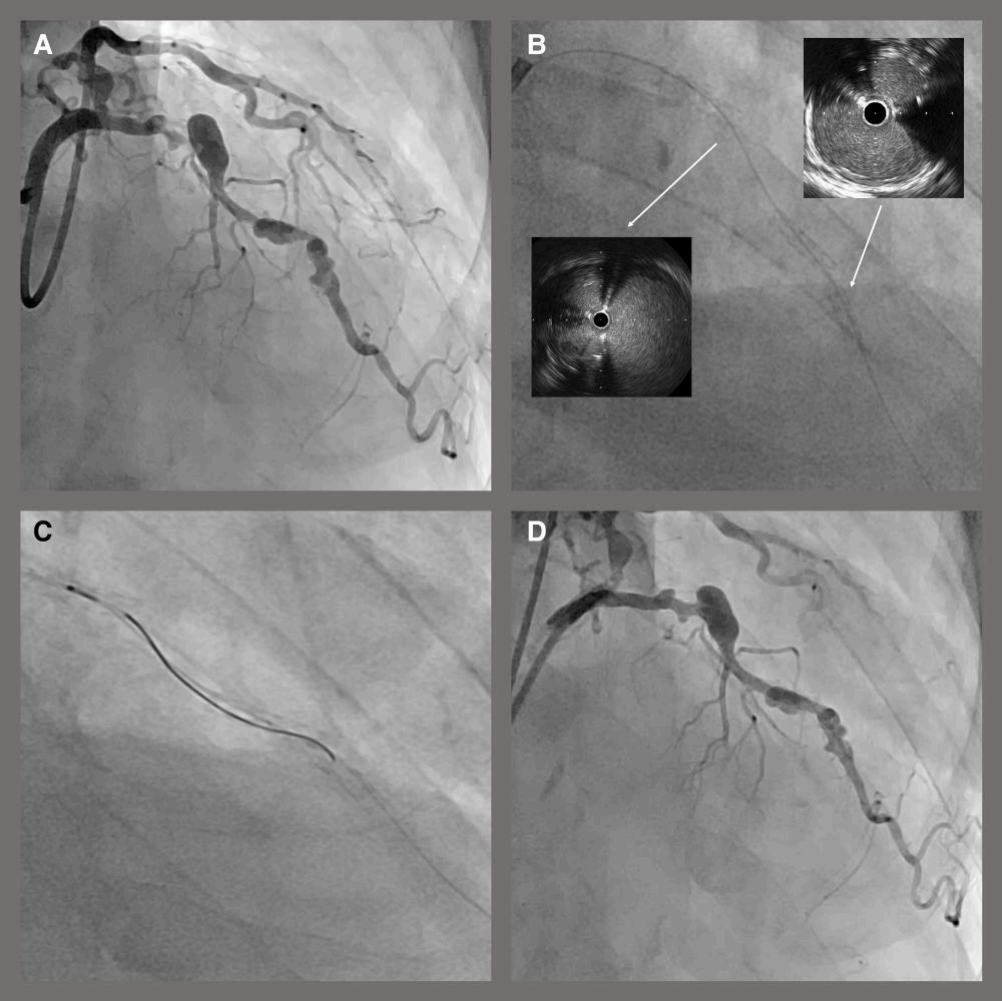
Percutaneous coronary intervention. (*A*) Initial coronary angiography. (*B*) Intravascular ultrasound revealed that the first guide wire was passing outside the stent in two places (arrows). (*C*) Rewiring using a double-lumen microcatheter and another guide wire was performed. Finally, intravascular ultrasound imaging confirmed that the guide wire had passed entirely inside the stents. (*D*) After drug-coated balloon dilatation, final angiography showed good result without significant stenosis.

We performed PCI via the left radial artery using a 6 Fr guiding catheter. Initial angiography is shown in *Figure 3A*. After placing the guide wire, IVUS revealed that the guide wire was passing outside the stents in two places (*Figure 3B*). Thus, rewiring using a double-lumen microcatheter with another guide wire was performed (*Figure 3C*). Finally, IVUS imaging confirmed that the guide wire passed entirely inside the stents. The ISR lesions were dilated with a 3 mm high-pressure balloon and 3 mm DCB dilation was subsequently performed. The final angiogram showed thrombolysis in myocardial infarction grade 3 flow without significant stenosis (*Figure 3D*). The patient was discharged after the procedure without any complications. His symptoms were relieved, and SPECT also showed improvement in ischaemia. His clinical course was uneventful and coronary computed tomography angiography after 1 year showed no change in the size of coronary artery aneurysms.

## Discussion

We report a case in which ISR, stent fractures, and coronary aneurysms were simultaneously diagnosed 14 years after SES implantation. In this case, since multiple coronary aneurysms were observed in the SES implanted sites, it is considered that coronary aneurysms occurred first due to hypersensitivity to the SES, which put a load on the malapposed stents and caused fracture, leading to restenosis. Previous study also speculated that coronary aneurysm could cause stent fracture.^3^

There is no established treatment for coronary aneurysms. Although surgical aneurysm repair is often reported, it is highly invasive and there are inoperable cases and high-risk cases. The covered stent is also well reported; it is not indicated for lesions with important side branches diverging next to the aneurysm site because side branch occlusion is inevitable. Stent-assisted coiling is useful for percutaneous treatment of coronary aneurysms with branches, but the procedure is complicated and carries potential risks of myocardial infarction due to coil migration and intraprocedural aneurysmal rupture due to coil or microcatheter. Moreover, there is a concern that re-stenting at the site of coronary aneurysm due to hypersensitivity reaction to the stent may enlarge the aneurysm due to further hypersensitivity reaction. Although there is no established diameter criterion for the decision to intervene in patients with coronary artery aneurysm, it has been proposed to consider invasive treatment for coronary aneurysm >20 mm in diameter.^4^

Given the patient background and lesion morphology, we treated the ISRs with DCB without intervention for coronary aneurysms. This is the first report of ISR with coronary aneurysm treated with DCB. The guide wire easily migrated outside the malapposed stents, which could be revealed by IVUS, not fluoroscopy. It was considered that the ISR lesions dilatation along the guide wire that passed outside the stents would lead to poor expansion of the lesions and worsening of the stent fractures. Therefore, we performed rewiring so that the guide wire passed completely inside the stents and confirmed it using IVUS. The risk of thrombosis and rupture due to the coronary aneurysms remains, so we plan to continue dual antiplatelet therapy and aneurysms diameter follow-up.

## Data Availability

All data will be shared on reasonable request to the corresponding author.

## Lead author biography

Takanari Fujita graduated from Hokkaido University School of Medicine in 2012. After completing 2 years of residency programme, he worked as a cardiology senior resident at Kyoto University Hospital for 2 years. Then, he trained as an interventional cardiologist at Koto Memorial Hospital for 4 years. He is currently working at the Department of Cardiovascular Medicine, Japanese Red Cross Wakayama Medical Center, Wakayama, Japan.

**Consent:** Written informed consent for submission and publication of this case report has been obtained from the patient in line with COPE guidance.

**Funding:** None declared.
